# Ensemble Federated Learning Approach for Diagnostics of Multi-Order Lung Cancer

**DOI:** 10.3390/diagnostics13193053

**Published:** 2023-09-25

**Authors:** Umamaheswaran Subashchandrabose, Rajan John, Usha Veerasamy Anbazhagu, Vinoth Kumar Venkatesan, Mahesh Thyluru Ramakrishna

**Affiliations:** 1 Department of Artificial Intelligence and Machine Learning, New Horizon College of Engineering, Bangalore 560103, India; dr.umamaheswaran.nhce@newhorizonindia.edu; 2 Department of Computer Science, College of Computer Science and Information Technology, Jazan University, Jazan 45142, Saudi Arabia; rsubbaiah@jazanu.edu.sa; 3 Department of Computing Technologies, School of Computing, Faculty of Engineering and Technology, SRM Institute of Science and Technology, SRM Nagar, Kattankulathur, Chennai 603203, India; anbuveera@gmail.com; 4 School of Computer Science Engineering and Information Systems, Vellore Institute of Technology, Vellore 632014, India; 5 Department of Computer Science and Engineering, Faculty of Engineering and Technology, JAIN (Deemed-to-Be University), Bangalore 560066, India

**Keywords:** lung cancer classification, diagnostics, federated learning models, thresholding, optimization, decentralized computation

## Abstract

The early detection and classification of lung cancer is crucial for improving a patient’s outcome. However, the traditional classification methods are based on single machine learning models. Hence, this is limited by the availability and quality of data at the centralized computing server. In this paper, we propose an ensemble Federated Learning-based approach for multi-order lung cancer classification. This approach combines multiple machine learning models trained on different datasets allowing for improvising accuracy and generalization. Moreover, the Federated Learning approach enables the use of distributed data while ensuring data privacy and security. We evaluate the approach on a Kaggle cancer dataset and compare the results with traditional machine learning models. The results demonstrate an accuracy of 89.63% with lung cancer classification.

## 1. Introduction

Lung cancer diagnosis and treatment utilizing computational approaches present multifaceted challenges at the intersection of medicine, data science, and technology. The etiology of lung cancers primarily involves somatic mutations arising from DNA resequencing events, induced by a myriad of factors, including environmental exposure and genetic predisposition. The detection of lung cancer at its early stages is hindered by the lack of distinct symptoms, often leading to delayed diagnosis and poor prognosis. Quantitative analysis by the World Health Organization (WHO) reveals a staggering global incidence of approximately 2.21 million reported cases of lung cancer annually, necessitating advanced research and innovative technological solutions to combat this prevalent and life-threatening disease. In the realm of computational methodologies, machine learning models have emerged as pivotal tools for addressing lung cancer challenges. These models are trained on diverse datasets, encompassing genomic profiles, radiological images (such as computed tomography scans), histopathological slides, and clinical records. Leveraging supervised and unsupervised learning paradigms, machine learning algorithms can discern complex patterns and features within these heterogeneous datasets, enabling early diagnosis, tumor subtyping, and survival prediction.

The successful amalgamation of computational methodologies with medical practices necessitates careful handling of medical terminologies and the implementation of standardized data representation schemas. Collaborative efforts among medical professionals, computational scientists, and domain experts are vital for constructing informative feature sets, minimizing data biases, and generating robust predictive models. State-of-the-art techniques like deep learning have exhibited exceptional capabilities in feature extraction and representation learning, empowering them to identify intricate biomarkers and genetic signatures, which were previously challenging to detect using traditional statistical methods. However, model interpretability remains a critical concern, as black-box models can hinder the medical community’s understanding of decisions made by these algorithms.

Translating computational results into clinical applications demands an adherence to rigorous validation and reproducibility standards. Cross-validation techniques, external validation cohorts, and robust statistical analyses are crucial to ensure model generalizability and clinical utility. Furthermore, establishing transparent reporting practices enhances the credibility and adoption of computational findings in the medical domain. Beyond diagnostic applications, computational approaches play a pivotal role in treatment monitoring and precision medicine. Predictive models can aid in drug sensitivity prediction, guiding oncologists to select personalized treatment regimens and optimize therapeutic interventions. Additionally, real-time monitoring systems can continuously assess treatment responses, enabling adaptive therapy and minimizing adverse effects.

Ethical considerations are imperative in the integration of technology into healthcare. Privacy preservation and secure data-sharing mechanisms are critical to safeguard patient data. Furthermore, continuous human oversight and the active involvement of medical professionals are essential to prevent overreliance on automated systems and ensure a patient-centered approach to care. The computational approaches, particularly machine learning models, hold tremendous promise in revolutionizing lung cancer diagnosis and treatment. By capitalizing on multidimensional data and leveraging cutting-edge algorithms, computational methodologies have the potential to usher in a new era of precision oncology, ultimately improving patient outcomes and transforming lung cancer management. However, a concerted effort among diverse stakeholders, rigorous validation, and ethical considerations are indispensable to unlock the full potential of computational technologies in combating lung cancer effectively.

### 1.1. Problem Statement and Research Motivation

The primary challenge inherent in this approach revolves around the notion of centralized processing. Existing machine learning models primarily rely on server-based computation and centralized data storage. Consequently, this leads to the accumulation of substantial amounts of data on the servers, resulting in anomalies during computation and training. The data processing and computation processes adhere to the functional requirements of a client-server architecture, meaning that decision-making heavily relies on local computational techniques and processes. Therefore, it becomes imperative to transition machine learning and computational models towards a decentralized server infrastructure to enable faster and broader training using diverse datasets. In a conventional peer-to-peer connected server setup, data collection, processing, and computation are confined to dedicated servers, thereby limiting access for extensive decision support. Moreover, when multiple servers are added to the baseline centralization or computation, the decision-making capabilities decrease, leading to a computational load overhead.

In this paper, we explore a Federated Learning (FL) approach for decision-making and the classification of lung cancer. We define and tailor FL models within a decentralized topology to facilitate faster and more secure computations. Additionally, we reframe this approach from a medical perspective. The decentralization of medical data aims to enhance processing and decision-making capabilities on a larger scale.

### 1.2. Objective and Contributions

This paper introduces a distributed federated network architecture for customizing local neural networks (NN) in Federated Learning (FL) models, with a focus on collective decision-making and storage in lung cancer datasets. The system ensures reliable computation and classification of lung cancer cases into normal, benign, and malignant categories. This classification relies on well-defined training and testing within a structured computational environment. This paper starts with an introduction outlining the rationale for the proposed approach in the context of FL and medical applications. A comprehensive literature review follows, summarizing recent developments in Federated Learning for medical data analysis, particularly lung cancer datasets.

The methodology section details the distributed federated cloud server’s technical aspects, including data streaming and processing mechanisms. It provides mathematical representations of customizing local NN models for lung cancer classification, likely employing optimization techniques such as federated averaging and differential privacy to ensure efficient training while preserving data privacy. This section also discusses data preprocessing, feature extraction, and model optimization within the distributed federated network. It may explore the convergence properties of federated optimization algorithms and the impact of network communication costs on system performance.

The major challenge of this approach is the concept of centralized processing. The approach of existing machine learning models is based on server computation and centralized data storage. This causes the overall servers to accumulate a large sum of data, causing anomalies in computation and training. The process of data processing and computation is typically based on the functional requirements of client server architecture and hence the aspect of decision-making is dependent on local computational techniques and processes. Hence, the machine learning cum computational models need to be shifted towards decentralized servers for a faster and wide range of training datasets. In the typical peer-to-peer connected server, the process of data collection, processing, and computation is bound to a dedicated server and hence provides limited and restricted access for wider decision support. The process is then made complex on adding multiple servers at baseline centralization or computation. The decision-making capabilities decrease and cause a load overhead for computations. In this paper, a Federated Learning (FL)-based approach is discussed for decision-making and classification of lung cancer. The FL models are defined and customized under a decentralized topology for faster and secure computations, whereas the approach is redefined in a medical perspective. The aspect of medical data under decentralization is to provide larger processing and decision-making capabilities.

This paper introduces a distributed federated network architecture aimed at thresholding and customizing local neural networks (NN) within the context of Federated Learning (FL) models. The main objective is to support collective decision-making and storage in the context of lung cancer datasets. The system ensures reliable computation and comparison of lung cancer cases, classifying them into normal, benign, and malignant categories. This classification process heavily relies on the training and testing of classifiers within a well-defined computational environment. This paper’s structure begins with an in-depth introduction, laying out the rationale for the proposed approach and its significance in the domain of FL and medical applications. A comprehensive literature review follows, highlighting the latest developments and findings related to Federated Learning models in the context of medical data analysis and diagnosis, particularly focusing on lung cancer datasets.

The methodology section delves into the technical intricacies of the distributed federated cloud server, elucidating the data streaming and processing mechanisms. This section provides a mathematical representation of how the customization and fine-tuning of local NN models are accomplished for lung cancer classification. Advanced optimization algorithms, such as federated averaging and differential privacy techniques, are likely employed to ensure efficient model training while preserving data privacy and security in the federated environment. The core mathematical representations offer detailed insights into the data preprocessing, feature extraction, and model optimization procedures within the distributed federated network. Special attention may be given to federated optimization algorithms’ convergence properties and the impact of network communication costs on the overall system performance.

In the results and discussion section, empirical findings are presented, showcasing the system’s performance on lung cancer datasets. The evaluation metrics employed might include accuracy, precision, recall, F1 score, and receiver operating characteristic (ROC) curves. In-depth analysis and comparison of the proposed approach against existing methodologies could further strengthen this paper’s technical content. The conclusion summarizes the key technical contributions of this paper, emphasizing the achieved advancements in lung cancer classification using the distributed federated network. The authors discuss the strengths and limitations of the proposed approach and suggest potential future research directions, such as refining the federated optimization algorithms or exploring different local NN architecture for improved performance. Overall, this paper contributes to the technical domain of FL in medical applications, particularly in the context of lung cancer diagnosis and classification. The integration of distributed computing, Federated Learning, and advanced mathematical representations demonstrates a rigorous and innovative approach to address the challenges posed by large-scale medical datasets while preserving data privacy and enabling collective decision support.

## 2. Related Work

Lung cancer is widely concerning for technical researchers to provide solutions, as early detection and categorization is minimal. From the technological front, solutions have been initialized from X-ray image processing and have evolved over time to Artificial Intelligence (AI). Various studies and observations for combating lung cancer detection, classification, and diagnosis have been recorded and published in the last decade. In [1,2], a systematic approach of a deep learning model is proposed on multiple data types such as X-rays, computed tomography (CT), and magnetic image resonance (MRI) images. The study focuses on how deep learning approaches can be implemented for lung cancer diagnosis and evaluation. Further, the approach toward lung cancer is based on the medical prospects in categorizing it as small cell lung carcinoma (SCLC) or non-small cell lung carcinoma (NSCLC) [3], to provide a wider perspective on occurrence and decision-making challenges. The study [3] concludes with a remark that neural networking algorithms are a much more reliable source of evaluation among researchers.

### 2.1. Initial Models

Machine learning models play vital role in understanding the behavioral approach of classifying and detecting lung cancers, with [4,5] proposing various models and techniques for optimizing lung cancer classification and decision-making. The studies have further provided a reliable understanding of the purpose and need for upgrading technological approaches in solving challenging issues such as carcinoma classification on normalized datasets. An advanced machine learning-based approach for lung cancer [6] is proposed for customization of improved images ranges and data types. The studies have included computer-aided design engineering (CADE) models for analyzing and validating datasets [7,8], to assure a reliable decision-making support. The computer-aided image systems and techniques provide a scalable environment for multi-objective dataset consideration and changes as per the technological development.

The lung cancer detection and prediction results and experience are shared and enhanced under a telemedicine ecosystem with an interdependency of electronic health records (EHR). The framework of Internet of Medical Things (IoMT) [9] has further provided an extended support for larger data sharing and decision-making. The terminology of Federated Learning (FL) provides greater prospects of shared information-based decision-making in a reliable manner. The federated models are reported by [10,11,12] in various medical data analysis and computations. The overall process of a Federated Learning model is to provide a distributed environment and a local- or client-based computation with reference to streamlining data operations. The architectural model and standard operation is proposed in [13]. The way forward for a Federated Learning model is to provide a threshold operation for customizing the information and data communication protocols via a remote server management tool.

### 2.2. Advanced Models

Deep learning (DL) models are used for the classification of lung cancer [14,15], with the classification based on the feature extraction of the lung cancer, while the techniques involved in the computation, such as the Histogram of Oriented Gradients (HoG), wavelet transformer-based features, and local binary patterns, are a few of the dominating approaches. Non-small cell-based lung cancer classification [16] is another prominent classification approach included in the domain of classifications followed by biomarkers [17]. The CT-based [18] classification under trivial approaches and the historiographic representations are included for reliable decision-making and support ecosystem development. This support system can be derived from contempory studies related to the classification process, as [19] with the extraction of patterns from the wave file and annotating them into depression and [20] with CT images classification-based on a fuzzy system.

Positron emission tomography (PET) and CT images are further considered for processing in a single environment to improve decision-making support, as in [21]. Ref. [22] provides a detailed survey and different types of lung cancer with respect to the imaging. The survey further assures that the dependency is improved from one systems operation and dataset to an independent computing unit. Approaches such as machine learning [23] and classification [24] provide justifiable decision-making capabilities on the lung cancer computation. These approaches further customize and process the behavioral model of computations algorithms [25,26]. The basic image computation and processing approach was defined and maintained on summarizing computational techniques, and hence the interdependency on decision-making was an unavoidable situation. The approach of trivial processing under centralized servers was replaced by distributed servers, with Federated Learning leading the domain. The Federated Learning (FL) models are based on the policies and standards of operation [27], with other architecture such as [28,29,30] under a decentralized server’s configuration. The approach benefits the operations and customization possibilities of processing lung cancer [31,32,33].

The studies in this survey discuss the application of various techniques, including machine learning and classification, for making informed decisions in lung cancer analysis. These techniques are tailored and refined to match the behavior of computation algorithms. Initially, simple image computation methods were used, but as the need for effective decision-making grew, more sophisticated approaches were adopted. The traditional method of processing data on centralized servers was replaced by using distributed servers with Federated Learning. This approach, known as Federated Learning (FL), relies on established operational policies and standards. It contrasts with other architecture like decentralized server configurations. FL offers advantages in the processing and customization of lung cancer-related operation.

## 3. Methodology

**Figure 1:**
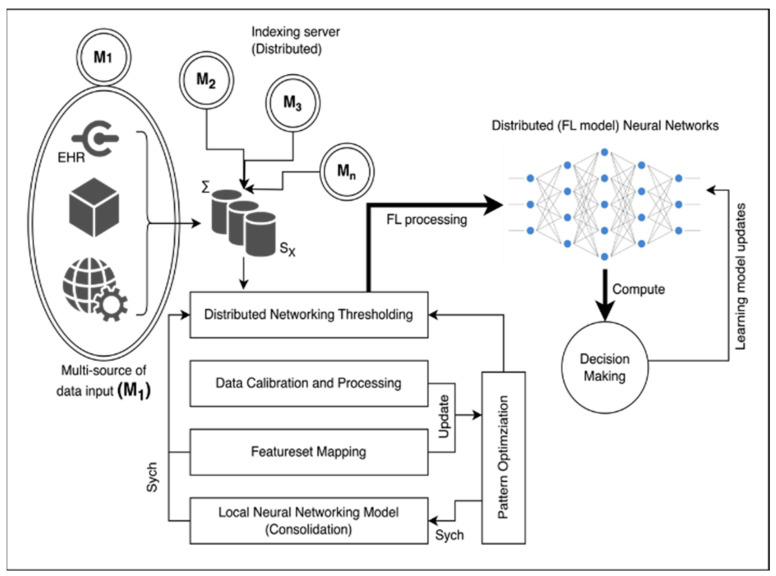
Architectural diagram of proposed technique.

The proposed methodology aims to establish a sustainable solution for tailoring data connectivity and transmission between different medical servers. This is achieved by leveraging available research and consultant data. The architecture of the proposed system is illustrated in Figure 1. At the core of the system, there are indexing servers which serve as foundational and highly reliable components of the system’s functioning. These indexing servers are denoted as (M). They are accompanied by a series of clusters of indexing servers originating from various sources and geographical locations. This collective assembly of server clusters forms the fundamental basis for implementing a Federated Learning approach. The centralized server (SX) acts as an aggregation point responsible for overseeing and coordinating the services provided by edge devices connected to the indexing servers (Mi). Typically, the indexing server (SX) is linked with a distributed networking threshold unit. This unit operates as an intermediate layer for various operations and assumes the role of a central decision-making and training package.

This entire process is facilitated by several key steps. Data calibration is carried out to ensure that the data being used are accurate and suitable for analysis. A process known as feature-set mapping is applied to correlate different data features appropriately. Moreover, a local neural network (NN) model is developed for each indexing server. This model is used to process the data effectively within each respective indexing server. The proposed methodology introduces a sustainable solution for customizing data connectivity and transmission among medical servers. This involves a complex but well-defined architecture where indexing servers play a pivotal role. The central server and distributed networking threshold unit contribute to the orchestration of services, while data calibration, feature-set mapping, and local neural network model development enhance the overall data processing procedure.

### 3.1. Dataset and Alignment Process

The dataset of non-small cell lung cancer (S0819) [31] is retrieved from the Cancer Image Archive (CIA) under multi-order cum multifunctionality cancer. The process of extracting a cancer (lungs) dataset from a larger repository is based on the feature-set mapping and alignment of the nearest-alike attribute on the grouping feature. Consider the dataset $\left(D\right)$ from CIA as the universal dataset and the fetched/selected dataset $\left(D_{C}\right)$ from the multi-order coordination as $\left(D_{C}\subseteq D\right)$ at a given instance. Consider $\left(D_{C}\Rightarrow D\right)$ at a generalized representation, whereas the process design is aligned to feature set $\left(F\right)$ as $\left(F\Rightarrow D_{C}\Rightarrow D\right)$ on the extraction. Typically, the orientation of information from one dataset pattern to another is related as $\left(\forall F\Rightarrow \forall F_{i}/i\in n\Rightarrow n\to \infty \right)$. The orientation resultant is computed, as shown in Equation (1).

(1) $$D_{C}=\underset{n\to \infty }{\lim}\left(\vec{F}\right).\frac{\delta \left(D_{C}\right)}{\delta t}$$

The limitation on the dataset $\left(D_{C}\right)$ is aligned with the features vector to assure reliable interaction and extraction of the dataset from $\left(D\right)$ at a given instance $\left(t\right)$. The extraction is further supported by the $\left(\Delta T\right)$ matrix for saturating the threshold in the dataset alignment process.

### 3.2. Multisource Indexing and Distributed Computation

**Figure 2:**
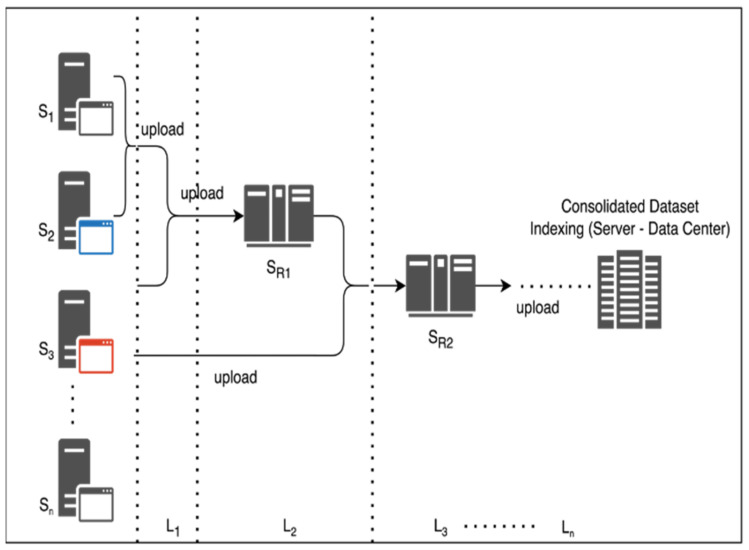
Hierarchical approach for server layering and consolidation.

The dataset $\left(D_{C}\right)$ is extracted and trained on localized servers $\left(S_{X}\right)$ for creating a multisource and multi-origin alignment. The processing of each server $\left(S_{i}\right)$ is dependent on routing servers $\left(S_{R}\right)$ such that, $\left\|\log S_{i}\Rightarrow S\right\|$ in a generic representation. The coordination alignment is shown in Figure 2. The hierarchy of each server $\left(S_{R}\Rightarrow S_{R1},S_{R2},S_{R3}\ldots S_{Rn}\right)$ with $\left(\forall S_{Ri}\Rightarrow S_{X}\right),$ with $\left(S_{X}\right)$ as the centralized server for distributed computing and $\left(S_{1},S_{2},S_{3}\ldots S_{n}\right),$ are in-line servers under coordination of ${\left(S_{R}\right)}_{i}$ as $\left(\forall S_{i}\subseteq S_{R_{i}}\right)$ and $\left(S_{i}\in S_{X}\right)$ on the operational setup. The server’s hierarchy assures the calibration and filtration of server-to-server interaction, and hence an ensemble Federated Learning model is generated. Typically, the process of data coordination and collection is from one centralized server to another, hence causing fractional losses. The proposed system is further structured and contributes complex-free computations.

The multisource Indexing and DNT is shown in Algorithm 1.
**Algorithm 1** Multisource Indexing and DNT
 Input: $\left(D_{C}\right)$ datasets on $\left(S_{X}\right)$ server alignment extracted and calibrated via IP address. Output: Process mapping and feature thresholding. Steps:
Fetching the hierarchy of servers $\left(S_{R}\Rightarrow S_{R1},S_{R2},S_{R3}.....S_{Rn}\right)$ such that, $\left(\forall S_{i}\subseteq S_{R_{i}}\right)$ and $\left(S_{i}\in S_{X}\right)$;**while**$\left(S_{i}\right)$Computing a distributed server $\left(S_{i}\right)$ such that, $\left(S_{i},S_{j}\in {\left(S_{R}\right)}_{i}\right)$**and while;**$∴S_{X}=\left\{\prod_{\left(i,j\right)\in k}^{\infty }\left(\frac{\delta \left(S_{i}\right)⊕\delta \left(S_{j}\right)}{\delta {\left(S_{R}\right)}_{k}}\right)\right\}$ computation on server operations and independencies;Compute feature $\left(F\right)$ such that, $\left(\forall F_{X}\Rightarrow \sum \left(S_{X}\cup S_{R}\right)\right)$Perform validation $\left(V\right)$ on extracted datasets $\left(D_{C}\right)$ to attain $\left(F\right)$ features;Generation of validation matrix $\left(R\right)$.

### 3.3. Distributed Network Thresholding (DNT) and Feature-Set Mapping

The process of the distributed network setup is to assure the monitoring of data losses and information breaches when computed in a distributed system. Typically, the server ${\left(S_{R}\right)}_{i}$ is responsible of each independent feature and hence the process of Distributed Network Thresholding (DNT) is introduced. The threshold acquires the value from the distributed server $\left(S_{i}\right)$ and is aligned with routing servers ${\left(S_{R}\right)}_{i}$, such that a common feature set is labeled from the ${\left(S_{R}\right)}_{i}$ as $\left(S_{i},S_{j}\in {\left(S_{R}\right)}_{i}\right)$ at a given instance of time, as shown in Equation (2).

(2) $$S_{X}=\left\{{\left(\frac{\delta \left(S_{i}\right)}{\delta t}⊕\frac{\delta \left(S_{j}\right)}{\delta t}\right)}_{\left(i,j\right)}\right\}\cup \left\{\underset{n\to \infty }{\lim}\left(\sum_{k=1}^{n}{\left(S_{R}\right)}_{k}\right)\right\}$$

(3) $$∴S_{X}=\left\{\prod_{i,j}^{\infty }\left(\frac{\delta \left(S_{i}\right)⊕\delta \left(S_{j}\right)}{\delta t}\right)\right\}\cup \left\{\underset{n\to \infty }{\lim}\left(\sum_{k=1}^{n}{\left(S_{R}\right)}_{k}\right)\right\}$$

(4) $$∴S_{X}=\left\{\prod_{\left(i,j\right)\in k}^{\infty }\left(\frac{\delta \left(S_{i}\right)⊕\delta \left(S_{j}\right)}{\delta {\left(S_{R}\right)}_{k}}\right)\right\}$$

According to Equations (3) and (4), the representation vector of multiple feature-set extraction from $\left(D\right)$ is future evaluated and represented. Since the feature-set coordination is a reliable entity, the grouping factor is aligned with the contributing factors. Hence, the generalized representation of $\left(S_{X}\right)$ can be represented as Equation (5).

(5) $$(i.e.,)\;S_{X}=\prod_{\left(i,j\right)\in k}\left\{\frac{\sum_{i}\left(\delta \left(S_{i}\right)\right)⊕\sum_{j}\left(\delta \left(S_{j}\right)\right)⊕....}{\delta {\left(S_{R}\right)}_{k}}\right\}$$

Thus, according to Equation (5), the representation matrix of multiple sources coordinated or calibration to a single source is studied and demonstrated. The incoming servers compute a feature $\left(F\right)$, as shown in Equation (6).

(6) $$F=\frac{\delta \left(S_{X}\right)}{\delta t}.\left\|\log{\left(S_{X}\right)}_{i}\right\|\cup \left\|\log\left(F_{X}\right)\right\|$$

where the feature $\left(F\right)$, computed with $\left(S_{X}\right)$ servers and its hierarchy, is recomputed with aligned threshold feature $\left(F_{X}\right)$, such that $\left(\forall F_{X}\Rightarrow \sum \left(S_{X}\cup S_{R}\right)\right)$ is on a multiple source and instance. Feature-set mapping is further computed by aligning the interdependent outcomes of multiple servers $\left(S_{X}\right)$ with the feature-set threshold $\left(F_{X}\right)$ accordingly, considering lung cancer features such as density, mass, orientation, and the weight are physical attributes cum features and the pixel ratio, pattern of growth, intensity of pixel, growth, and density of pattern expansion are a few of the digital computational parameters. Consider the physical attributes as mandatory parameters for recognition and validation $\left(P_{a}\right)$ and digital parameters $\left(P_{d}\right)$, such that a common fitting value on threshold is extracted.

Consider the validation $\left(V\right)$ as a functional vector for regional computations to acquire $\left(P_{a}\right)$ and $\left(P_{d}\right)$, respectively, then the representation is $\left(\forall V\Rightarrow P_{a}⊕P_{d}\right)$, such that the validation matrix $\left(R\right)$ is shown in Equation (7).

(7) $$R=\left\|\log_{n}\left(V\right)\right\|⊕\left\{\underset{n\to \infty }{\lim}\left(\frac{\delta \left(V\right)}{\delta t}\right)\right\}$$

(8) $$∴R=\left\|\log_{n}\left(V\right)\right\|⊕\left\{\underset{n\to \infty }{\lim}\left(\sum_{p_{a}}^{}\sum_{p_{d}}^{}\left(V_{\left(p_{a},p_{d}\right)}\right)\right)\right\}$$

Thus, according to the process of $\left(P_{a}\right)$ and $\left(P_{d}\right)$, the contribution matrix $\left(R\right)$ stores the rational values of feature threshold extracted.

### 3.4. Federated Neural Networking Computational Model

The federated neural network computational model is defined and correlated on the earlier prospects of vector validation $\left(V\right)$ via recognition and validation of $\left(P_{a}\right)$ and digital parameter $\left(P_{d}\right)$. The process of the computation model extracts the contribution matrix $\left(R\right)$ and further computes a rational thresholding under larger segmented values. Consider the validation matrix $\left(R_{X}\right)$ as $\left(R_{X}\subseteq R\right)$ under the considered dataset $\left(D_{C}\right)$. The attribute learning of Federated Learning is shown in Figure 2. Thus, according to Figure 2, the federated computation of the distributed cloud/server neural networks is optimized and processed. The independent server/clouds are connected via a self-common agreed firewall system cum configuration for indexing and data sharing. Typically, the follow-up server computes the local neural networking model to assure attributes optimization and minimization of computing indexes. The orientation model of the computational local neural networking cluster from multiple clouds defines the optimized attribute graphs. Typically, the interconnected servers are aligned time-to-time based on the source synchronization standards and cloud service providers.

### 3.5. Experimental Setup and Configurations

The objectives of extracting multi-order lung cancer classification are via the federated setup of cloud/server models. The federated cloud is a distributed cloud model of connecting remote clients via a centralized server for optimized data transfer. The federated approach in biomedical aspects plays a vital role for electronic health records (EHR) customization and remote accessing. The process of datasets (CT/CIA images) are distributed via federated cloud cluster $\left(fc_{1},fc_{2},fc_{3}\ldots \ldots \right)$, such that $\left(\forall fc_{i}\Rightarrow F\right)$ and the data in $\left(fc_{i}\right)$ are $\left(\exists F\right)$ (i.e.,) accessible to F. The data privacy and originality is highly preserved at federated configuration, and thus extracting the recommendation models of EHR patterns and attributes can be achieved at a faster rate.

The experimental setup is aligned using multi-operating system-based virtual machines and kubernetics alignment of cluster management. The server (master) and remote server (client) are aligned as per the database exchange norms for connectivity and coordination. The orientation of cancer attributes from a larger perspective are further considered and processed as the reconfigured thresholding attribute. The dataset is extracted from CIA liberty and further processed and cross-validated on customized data labels.

### 3.6. Implementation Details

The input CT lung cancer datasets are based on 50 low-dosage and pre-recorded lung cancer cases with 1.25 mm slice thickness. The dataset is processed with 60/40 training/training ratio for accuracy detection. The setup was a defined and calibrated platform of MATLAB 2018 with CPU i5 of 16 GB.

## 4. Results and Discussions

The classified datasets are further processed and customized using standard CT datasets and compared with NN, SVM, KNN, and DNN for local neural networking computation. The lungs’ CT datasets are classified and labeled as normal, benign, and malignant. The normal CT defined is unconditional images with no positive Region of Interest (RoI) features and attributes. The benign CT has a positive RoI on feature, whereas providing no harm or radiant growth to the lung cancer contribution and the malignant categorization is a positive and active representation of cancer growth; thus, the training and testing model is shown in Table 1 and Table 2, respectively and Table 3 depicts the Performance matrix validation of FL model on server nodes.

**Figure 3:**
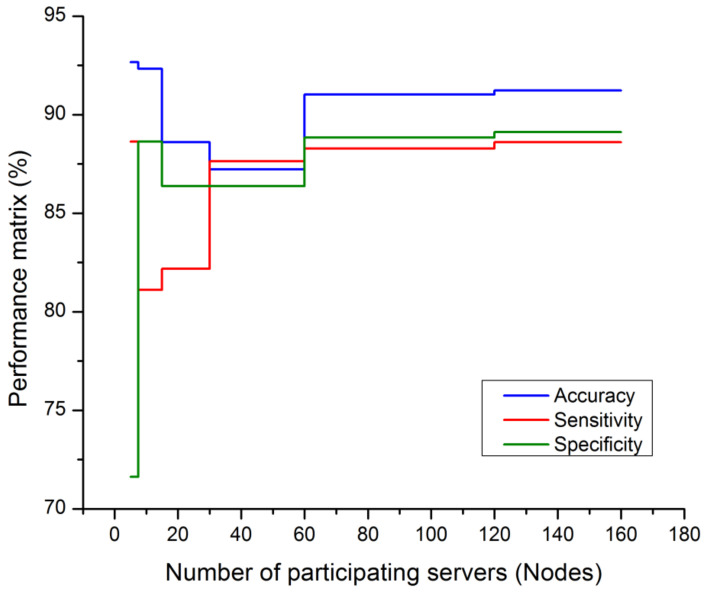
Performance matrix on participating nodes of FL model.

**Figure 4:**
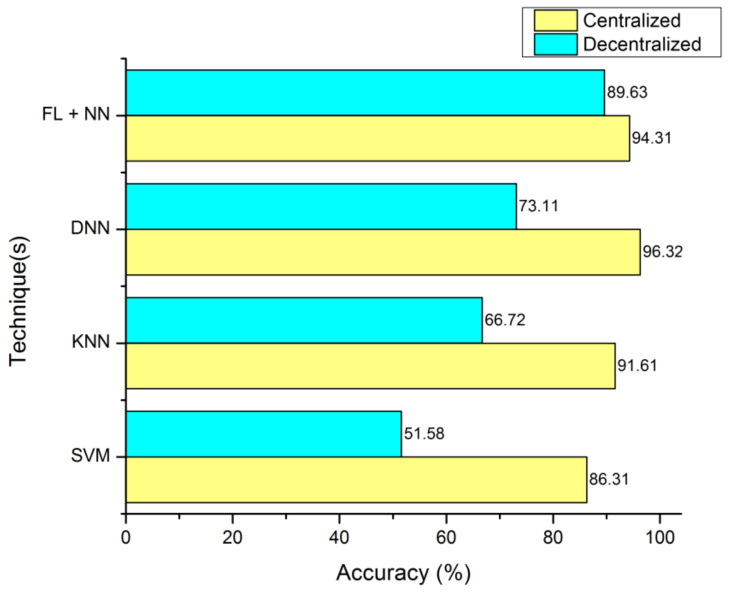
Accuracy computation between centralized server and decentralized server organization.

**Figure 5:**
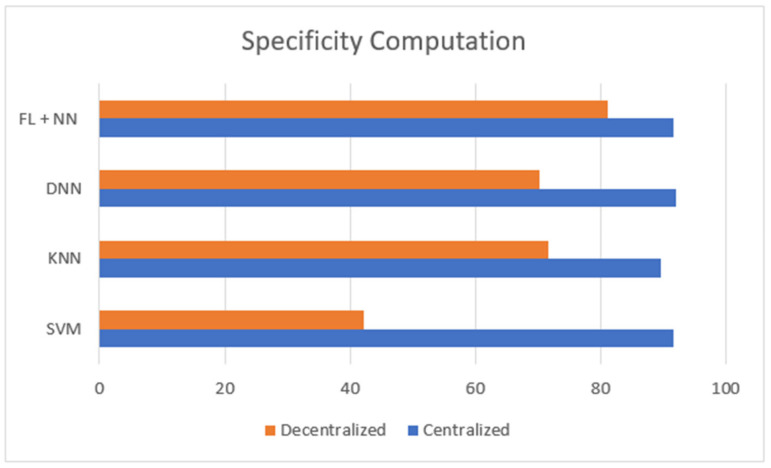
Comparison of specificity computation between centralized and decentralized server-based classification of lung cancer dataset.

Figure 3 is defined with the legend of performance matrix with respect to the participating servers used in costuming the data transmission and channeling via FL models. The indexing servers (S_X_) participating nodes are incremented in a series order of doubling. The nodes’ (servers) computational performances are estimated with accuracy, sensitivity, and specificity. The evaluation matrix is represented in Table 4 for detailed comparison with existing approaches and techniques. The proposed FL + NN has a demonstrative accuracy of 89.63%, in a decentralized approach, which is comparatively higher than other techniques, with the detail represented in Figure 4 and Figure 5, respectively.

## 5. Conclusions

The proposed technique was designed and developed based on the Federated Learning model of decentralized servers used for computational decision-making lung cancer classification. Typically, the cancer dataset is trained and tested with a trivial centralized cloud and a setup of federated distributed cloud. This approach has successfully trained on 60:40 ratios via multi-order attributes and features. This process is attained with a thresholding of dataset features from distributed computing to derive a threshold of feature-set mapping. This approach has been successfully validated on distributed computational local neural networks for data communication and calibration for lung cancer classification via a federated model. The approach demonstrated on the FL-NN setup had an accuracy of 89.63% under the federated decentralized technique. This approach can be further developed on multidimensional medical models and electronic health records (EHR) to provide a reliable recommendation and decision support system.

## Figures and Tables

**Table 1:** Training and testing model for centralized server classification.

Phase	Server Configuration	Dataset Type	Classifier			
			Normal	Benign	Malignant	Total
Training	Centralized Servers (Cloud/Server Model)	Normal	20	06	12	38
		Benign	02	18	06	26
		Malignant	06	04	22	32
		Total	28	28	40	96
Testing	Centralized Servers (Cloud/Server Model)	Normal	12	02	00	14
		Benign	18	11	04	33
		Malignant	00	12	06	18
		Total	30	25	10	65

**Table 2:** Training and testing model for decentralized server classification (federated model).

Phase	Server Configuration	Dataset Type	Classifier			
			Normal	Benign	Malignant	Total
Training	Decentralized Servers (FL Model)	Normal	20	04	14	38
		Benign	06	13	07	26
		Malignant	09	07	26	42
		Total	35	24	47	106
Testing	Decentralized Servers (FL Model)	Normal	16	03	00	19
		Benign	12	18	04	34
		Malignant	06	13	18	37
		Total	19	34	22	90

**Table 3:** Performance matrix validation of FL model on server nodes.

Number of Participating Servers (Nodes)	Accuracy (%)	Sensitivity (%)	Specificity (%)
5	92.67	88.63	71.62
10	92.33	81.11	88.64
20	88.61	82.18	86.38
40	87.23	87.63	86.37
80	91.03	88.28	88.84
160	91.23	88.61	89.12

**Table 4:** Comparative model of computational matrix.

Technique(s)	Centralized			Decentralized		
	Accuracy (%)	Sensitivity (%)	Specificity (%)	Accuracy (%)	Sensitivity (%)	Specificity (%)
SVM	86.31	91.62	71.66	51.58	42.3	31.66
KNN	91.61	89.67	88.62	66.72	71.62	66.11
DNN	96.32	92.11	90.72	73.11	70.32	81.68
FL + NN	94.31	91.66	88.62	89.63	81.26	80.31

## Data Availability

The dataset used for the findings is included in this manuscript.
